# Features of the Liver Microstructural Organization of the Bactrian Camel

**DOI:** 10.3390/ani15192921

**Published:** 2025-10-08

**Authors:** Lyalya Musina, Anna Lebedeva, Ludmila Drozdova, Alexey Prusakov, Vladimir Ponamarev

**Affiliations:** 1 Russian Eye and Plastic Surgery Centre, Bashkir State Medical University, 450008 Ufa, Russiajeol02@mail.ru (A.L.); 2 Department of Morphology and Expertise, Ural State Agrarian University, 42 Karl Liebknecht Str., 620075 Yekaterinburg, Russia; 3 Department of Internal Medicine of Animals, Saint-Petersburg State University of Veterinary Medicine, 196084 Saint Petersburg, Russia; 4 Department of Pharmacology and Toxicology, Saint-Petersburg State University of Veterinary Medicine, 196084 Saint Petersburg, Russia

**Keywords:** camel, liver, ultrastructure, hepatocytes, lipid metabolism

## Abstract

**Simple Summary:**

Camels exhibit remarkable physiological adaptations that enable them to survive in harsh, resource-limited environments. A key factor in this resilience is their unique fat metabolism, which has a profound influence on the structure and function of their internal organs. This study specifically investigated the liver of the Bactrian camel. Through detailed electron microscopic analysis of liver tissue samples, researchers discovered several distinctive features. Unlike the livers of many other mammals, the camel liver possesses exceptionally large hepatocytes (25–30 µm). It may be related to the need to store metabolites and water. A further significant finding was the presence of fewer (registered in 12–15% of all hepatocytes), medium-sized (0.5–2 µm) lipid droplets in the hepatocytes, compared to the other species. Large numbers (for Kupffer cells, 15–20 cells per 10,000 µm^2^) and higher activity of the specialized cells (Ito and Kupffer) aimed towards liver protection and immune system activation. In summary, this research demonstrates that the camel liver has evolved specialized structural and cellular adaptations that directly support its survival in extreme conditions.

**Abstract:**

Camels have developed unique adaptive mechanisms, one of which is the active accumulation of lipids. This metabolic feature has a direct influence on the liver ultrastructure. Its analysis reveals how exactly the hepatocytes have evolved to effectively store fat and neutralize toxins, which is crucial for survival in the desert. Considering the latter, the aim of this research is to establish the features of the microstructural organization of the liver of the Bactrian camel (*Camelus bactrianus*). This study was conducted using 15 liver tissue fragments from 5 healthy Bacterian camels (3 pieces from each animal) via biopsy. The sections were examined using a JEM-1011 electron microscope at magnifications of 2500–8000. Electron microscopic analysis of hepatocytes revealed a significantly larger hepatocyte diameter (25–30 µm), suggesting an adaptation for metabolites and water storage. Hepatocytes exhibited fewer, medium-sized (0.5–2 µm) lipid droplets, present in only 12–15% of cells. A high density of specialized Kupffer (15–20 cells per 10,000 µm^2^) and activated Ito cells was observed, indicating enhanced detoxification and immune functions. These specific ultrastructural features provide a model for studying metabolic resistance and inform veterinary diagnostics and husbandry practices for this species.

## 1. Introduction

Camel farming is predominantly developed in the steppe and semi-desert regions of eastern Central Asia, Mongolia, and neighboring territories of Russia and China [1,2,3]. This is due to the fact that the camel, as a species of livestock, successfully combines high meat and dairy productivity, as well as working capacity [2,4]. Camels have adapted to living in a strongly continental, dry climate with hot and dry summers and very cold and snowy winters [5,6,7]. In their natural habitat, they often face a lack of food and water. During the evolutionary development, these circumstances led to the emergence of various types of adaptive mechanisms in the organs of these animals [8,9,10]. One of them is fat accumulation. Fat is a reserve supply of nutrients, energy, and water. Its accumulation in certain parts of the organism prevents excessive excretion of water. The liver is directly involved in fat metabolism since it synthesizes cholesterol and other forms of lipoproteins [11]. Features of fat metabolism, manifested in increased secretion and accumulation of lipids, inevitably have a direct impact on its structure [6,12,13,14]. Considering the latter, the aim of this research is to establish the features of the microstructural organization of the liver of the Bactrian camel (*Camelus bactrianus*).

## 2. Materials and Methods

The studies were conducted using liver tissue fragments obtained during the planned slaughter of five 2.5-year-old Kazakh camels (3 male, 2 female) raised on a private farm in the Astrakhan Region. The experimental animals were characterized by good and high body condition, which corresponded to categories B and C on the generally accepted body assessment scale for camels [15]. The average body condition score was 3.5 points (on a 5-point scale), indicating no signs of undernourishment or obesity. The muscle mass was well developed, the ribs were not visualized but easily palpated, and fat deposits in the hump and body area were moderately expressed.

Thirty days before the planned slaughter, the animals were transferred to a standard maintenance diet used on the farm in order to stabilize metabolic processes and eliminate the impact of stress associated with a sharp change in feeding. The diet consisted of alfalfa hay, barley, and wheat bran with constant access to water and table salt. The purpose of this protocol was not intensive fattening to increase live weight but to ensure a physiological feeding rate corresponding to the needs of a clinically healthy adult animal. This made it possible to obtain representative tissue samples reflecting the normal physiological state of the organ. All camels underwent a mandatory veterinary examination before being included in the experiment. Inclusion criteria were no history of chronic systemic diseases and normal body temperature (within 36.5–38.5 °C), heart rate (45–55 bpm), and respiration (8–16 bpm), as well as appetite and behavior. Animals were clinically observed daily. At the time of slaughter, all camels were considered clinically healthy: visible mucous membranes were pink in color, and there were no signs of diarrhea, cachexia, jaundice, or other pathological conditions.

After slaughter, a general macroscopic examination of the carcasses and internal organs was carried out. The liver of each animal was removed, weighed, and subjected to detailed external and cross-sectional examination. The consistency of the organ was elastic, and the edge was sharp. The color was a uniform dark brown with a characteristic smoked shade, typical of camels [16,17,18]. The cut surface was smooth and moist, with a clear and well-defined lobular architectural pattern. Visible portal tracts and walls of large vessels without signs of compaction or pathological dilation. The gallbladder and extrahepatic bile ducts were not dilated; the mucous membrane of the gallbladder had no ulcerations.

During the macro- and microscopic examination, special attention was paid to identifying possible parasitic lesions. No macroscopically visible cysts, abscesses, nodules, fibrous strands, or adult helminths were found on the liver capsule and parenchyma, as well as in the lumen of the bile ducts, indicating the absence of acute or chronic parasitic infestations (e.g., fascioliasis, echinococcosis, or dicrocoeliosis) at the time of slaughter.

For electron microscopy, immediate and adequate tissue fixation is critical to prevent the occurrence of ultrastructural artifacts (mitochondrial swelling, membrane destruction, etc.). A special trocar for electron microscopy allows a very small tissue sample (biopsy) to be collected and immediately immersed in a fixative (usually glutaraldehyde) with minimal mechanical trauma and time delay. The Menghini method was used [19,20].

To ensure maximum preservation of the ultrastructure, three small pieces of tissue (approximately 1 × 1 × 3 mm) from each animal were collected from an identical area of the right main lobe of the liver and immersed in a fixative within no more than 3–5 min after exsanguination of the animal. Such a short interval is critical for preventing postmortem autolytic changes [21].

Further processing of selected tissue samples for the purpose of producing ultrathin sections was carried out following generally accepted methods [22]. Primary fixation was performed by immersing the samples in cold (4 °C) 2.5% glutaraldehyde solution in 0.1 M cacodylate buffer (pH 7.2–7.4) for at least 24 h. After washing in the same buffer, the material was post-fixed for 2 h in 1% osmium tetroxide (OsO_4_) solution in the same buffer.

After dehydration in alcohols and acetone and embedding in epoxy resin (araldite), semi-thin sections of 1–2 µm thickness were produced on an ultratome (Leica UC7, Wetzlar, Germany) for light-optical control. Then, serial ultra-thin sections with a thickness of 70–90 nm were produced from the blocks. The sections were examined using a Jem-1011 electron microscope (JEOL, Tokio, Japan) at magnifications of 2500–8000. When designating cellular and non-cellular structures, the terminology corresponding to the International Histological Nomenclature was used [23].

This study complied with the basic requirements set out in the “Rules of Laboratory Practice” in accordance with the Order of the Ministry of Health and Social Development of the Russian Federation dated 23 August 2010 No. 708n “on approval of the Rules of Laboratory Practice”. Ethical principles for the treatment of animals were observed in accordance with the “European Convention for the Protection of Vertebral Animals Used for Experimental and Other Scientific Purposes. cets no. 123.” All manipulations with the animals were carried out in accordance with the ethical principles approved by the Ethics Committees at the Russian Eye and Plastic Surgery Centre of the Bashkir State Medical University, Federal State Budgetary Educational Institution of Higher Education “Ural State Agrarian University” and Federal State Budgetary Educational Institution of Higher Education “Saint-Petersburg State University of Veterinary Medicine”.

## 3. Results

**Figure 1:**
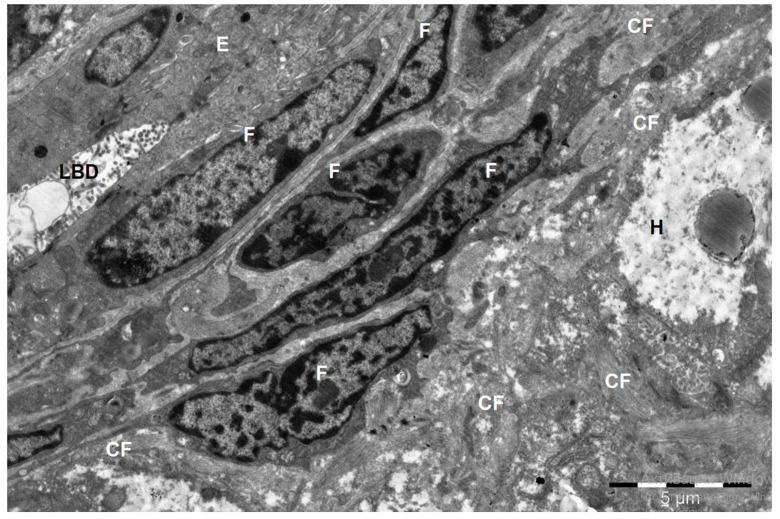
Fragment of the portal tract of the camel liver. LBD—lumen of the bile duct; E—bile duct epithelial cells; F—fibroblast cells; CF—collagen fibers; H—fragment of a hepatocyte with a lipid droplet in the cytoplasm. Electron microphotography.

**Figure 2:**
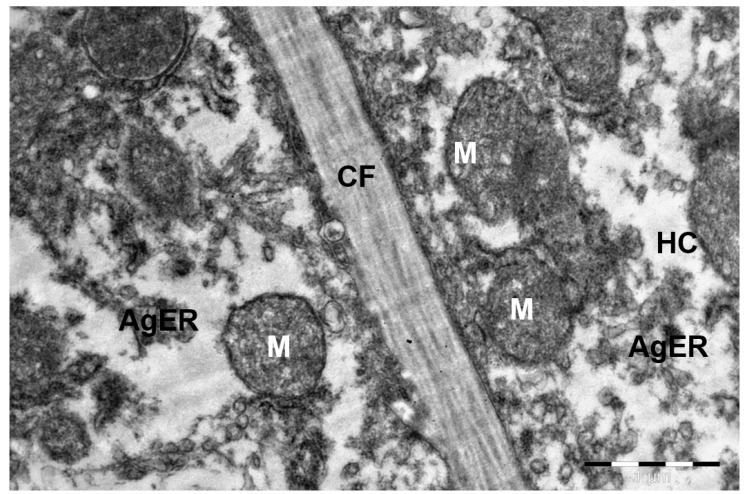
Collagen fibers between the lateral surfaces of the hepatic lobules in a camel liver. HC—hepatocyte cytoplasm; M—mitochondria; AgER—agranular endoplasmic reticulum channels; CF—collagen fibers between the liver lobules. Electron microphotography.

It was established that in the camel, the portal tracts lay predominantly in the area of the lateral edges of the lobules adjacent to each other and were formed by the interlobular arteries and veins, as well as the interlobular excretory bile duct. During histological examination, it was noted that the liver lobules of the studied animals have a prismatic shape and are clearly delimited from each other by a layer of connective tissue, which was confirmed by electron microscopic studies. The connective tissue reached its greatest thickness in the area of the portal tracts and its least development in the area of contact of the lobules with each other by their lateral surfaces (Figure 1 and Figure 2).

**Figure 3:**
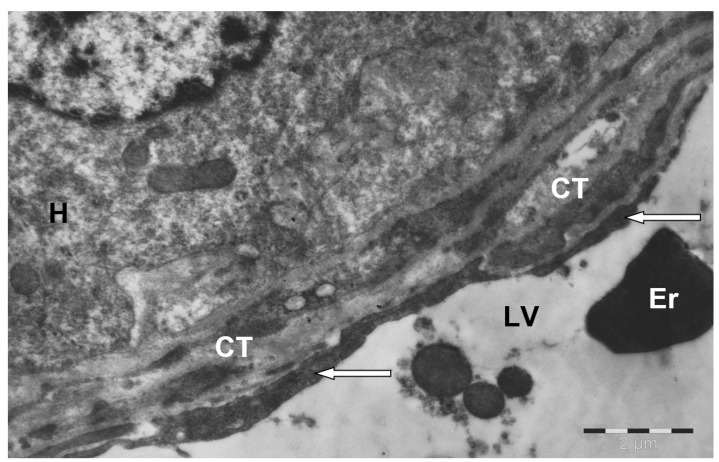
Annularly arranged bundles of connective tissue (CT) around the central vein in the camel liver. LV—lumen of the central vein; H—hepatocyte; endothelial cells (↑) of the vein wall; Er—erythrocyte in the lumen of the vein. Electron microphotography.

The connective tissue separating the lobules gave rise to connective tissue strands, which thinned as they moved toward the center. In turn, the central vein lay surrounded by ring-shaped bundles of connective tissue, penetrated by sinusoidal capillaries flowing into it (Figure 3).

The bulk of the camel liver parenchyma was formed by hepatocytes, which were relatively large (about 25–30 µm in diameter) epithelial cells of a round or polygonal shape. Electron microscopy showed that their cytoplasmic membrane was represented by two layers—outer and inner. They were separated by a light osmiophobic space, the width of which varied between 2.0 and 3.0 nm. Lining up in rows, hepatocytes formed hepatic beams, in the center of which there was a space for the accumulation and excretion of the bile they produced—the bile capillary. Thus, one pole of the cells—biliary—was directed into the lumen of the bile capillary formed by them, and the other, vascular, adjoined the wall of the sinusoidal capillary. The specified topographic location causes differences in the ultrastructural organization of these cell parts, determined by the function performed.

**Figure 4:**
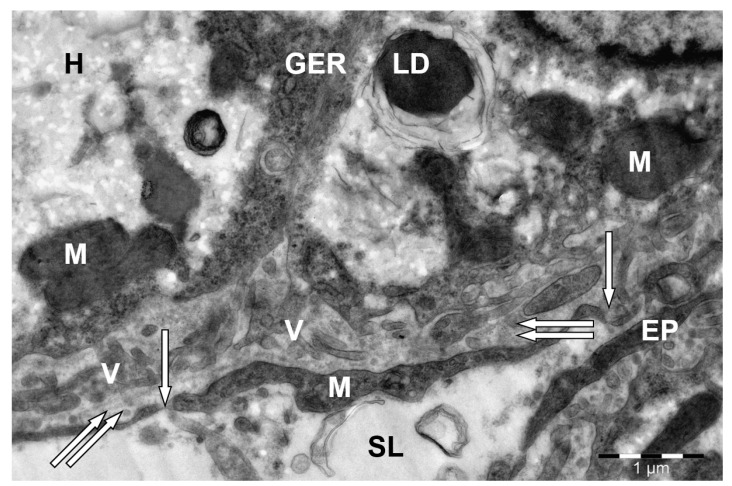
Vascular (sinusoid) side of a hepatocyte (H) in camel liver. V—hepatocyte villi; M—mitochondria; GER—granular endoplasmic reticulum channels; LD—lipid droplet; EP—endothelial cell processes; endothelial cell pores (↑); perisinusoidal space of Disse (↑↑); SL—sinusoid lumen. Electron microphotography.

The vascular (sinusoid) side of the hepatocytes facing the lumen of the hemocapillary formed numerous microvilli of varying length (Figure 4). This side of the hepatocytes and the endothelial cells lining the entire surface of the sinusoid capillaries and their long processes formed a space called the Disse space.

**Figure 5:**
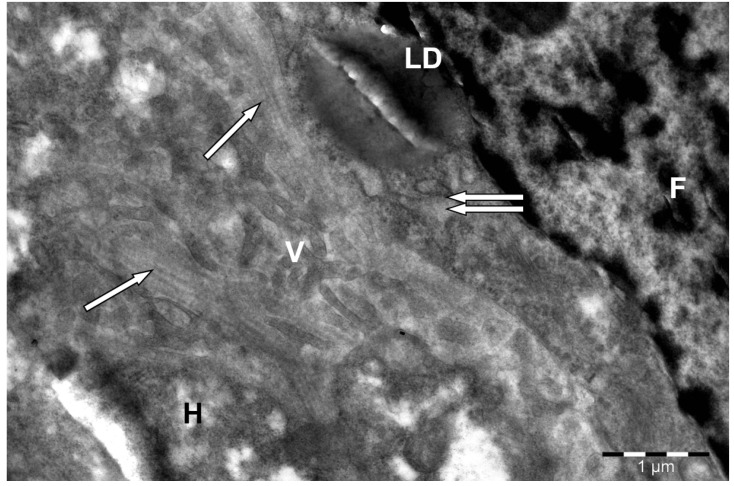
Sinusoidal side of a hepatocyte (H) in camel liver. F—fibroblast; dilated channels of the granular endoplasmic reticulum (↑↑); LD—lipid droplet; V—hepatocyte villi; collagen fibers (↑) in the perisinusoidal space of Disse. Electron microphotography.

Short microvilli were located mainly in the presinusoidal space, while long ones penetrated it and, passing through the pores of the sinusoid endothelial cells lying on the discontinuous basement membrane, penetrated into its lumen, directly contacting the blood. In many areas of the Disse space, a three-dimensional network of thin collagen fibers was detected together with fibroblasts (Figure 5). The thickness of collagen fibers of connective tissue (Disse’s space) in a camel is about 1.2–1.5 μm, which is approximately 1.5–2 times greater than in most mammals (0.7–0.9 μm).

In some areas, not only the processes of the sinusoid endothelial cells but also their elongated, large bodies containing a large, flattened nucleus were visible in the field of view. The cytoplasm of endothelial cells contained ribosomes and polyribosomes, single short cisterns of the granular endoplasmic reticulum, small oval mitochondria, and a large number of vesicles and bubbles.

**Figure 6:**
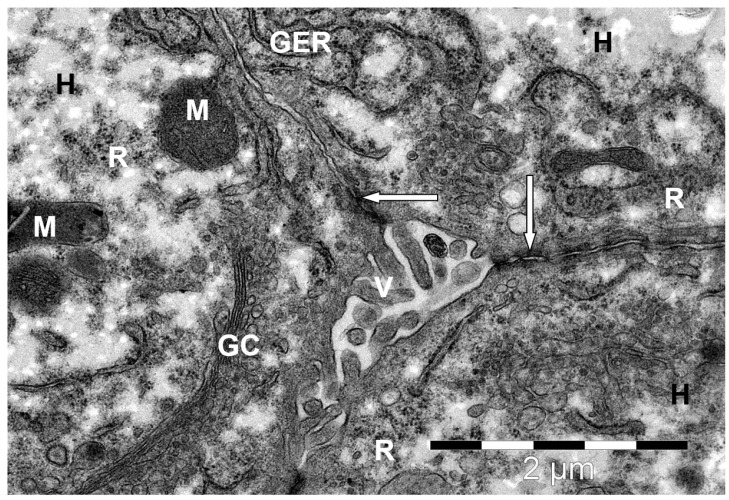
Bile canaliculi with villi (V) on the biliary side of a camel liver hepatocyte. H—hepatocyte; desmosomes (↑); GC—Golgi complex; M—mitochondria; GER—granular endoplasmic reticulum channels; R—ribosomes and polyribosomes. Electron microphotography.

The opposite (biliary) surface of the hepatocytes, facing the bile capillary, formed many microvilli facing its lumen (Figure 6). Very often, in the cytoplasm of hepatocytes near the bile canaliculi, in addition to long cisterns of granular endoplasmic reticulum, free ribosomes, and polyribosomes, the Golgi complex was clearly visible in the form of flattened cisterns arranged in parallel stacks with numerous vesicles surrounding them. This probably indicates the participation of the described organelle in the synthesis of bile components.

It should be noted that the lateral parts of the liver cells near the bile capillary were in contact with each other via dense osmiophilic desmosomes, which provided increased strength of the intercellular connection.

**Figure 7:**
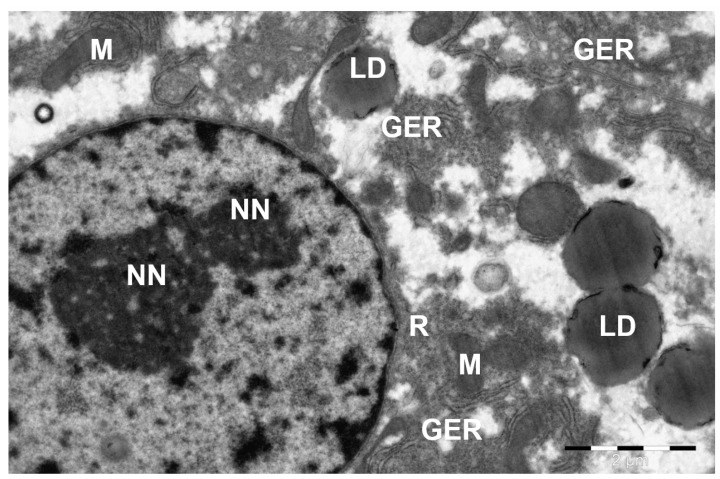
Structure of a camel liver hepatocyte. NN—nucleolus in the nucleus; LD—lipid droplet; M—mitochondria; GER—granular endoplasmic reticulum channels; R—ribosomes and polyribosomes. Electron microphotography.

The nuclei of the hepatocytes were sometimes located in the center, sometimes eccentrically. Their position also depended on the amount of inclusions accumulated in the cytoplasm, especially of a lipid nature. The size of the nucleus corresponds to the general species norm for large mammals (about 7–10 µm) and has no critical differences, since its function (transcription) is universal. Two layers separated by a light strip of perinuclear space represented the nuclear membrane. The nuclear matrix was electron-light in its composition. Inclusions of finely dispersed euchromatin were distinguishable. At the same time, heterochromatin in the form of small conglomerates was concentrated along the inner nuclear membrane. The sizes of the hepatocyte nuclei varied, with one or two electron-dense nucleoli being detected in almost every one of them (Figure 7).

In addition to round and oval mitochondria with thin cristae and dark mitochondrial matrix, elongated thin cisterns of granular and short bubble-shaped tubules of smooth endoplasmic reticulum and accumulations of glycogen and lipid droplets were detected in the cytoplasm of hepatocytes. Very small glycogen granules in the cytosol of hepatocytes were both scattered and grouped, but most often surrounding fat droplets. In the case of accumulation of lipids in the cytoplasm of hepatocytes, as a rule, many large lipid droplets were detected. The proportion of hepatocytes containing lipid droplets averages 12–15%, which is significantly lower than, for example, in laboratory rats (35–40%) or domestic cattle (25–30%), indicating adaptive resistance to lipotoxicity.

**Figure 8:**
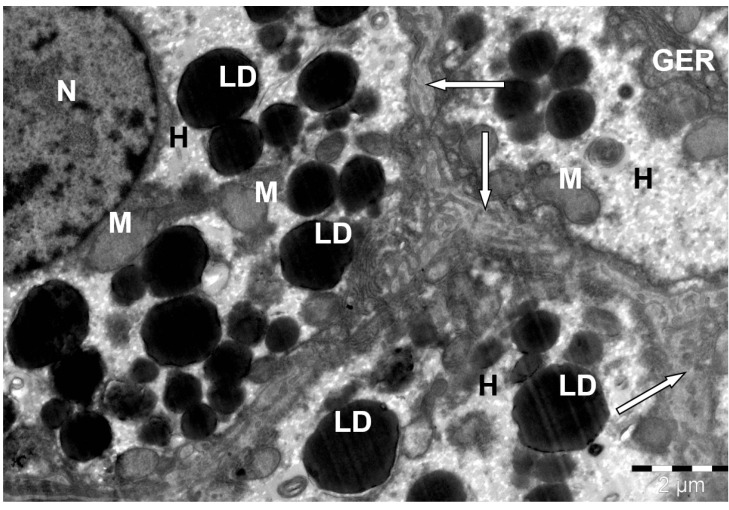
Structure of a camel liver hepatocyte. H—hepatocyte; N—nucleus; LD—lipid droplets; M—mitochondria; GER—granular endoplasmic reticulum channels; sinusoidal capillaries (↑). Electron microphotography.

Sometimes hepatocytes with small and medium-sized fat droplets were found, and these inclusions were located predominantly in the cytoplasm of the sinusoid pole of hepatocytes (Figure 8). It should also be noted that their accumulation occurred most intensively in the hepatocytes of the peripheral and middle zones of the liver lobules, rather than in their center.

**Figure 9:**
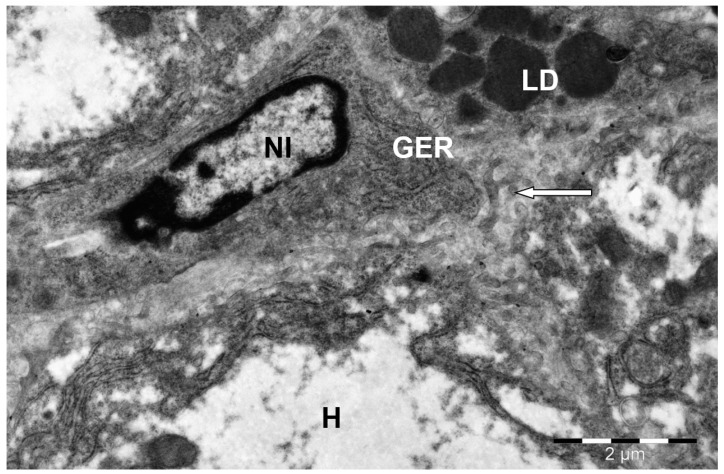
Fat-storing Ito cell in the camel liver. H—hepatocyte; NI—Ito cell nucleus; LD—lipid droplets; GER—granular endoplasmic reticulum channels; hepatocyte villi in sinusoids (↑). Electron microphotography.

In the liver of the animals studied, perisinusoidal fat-accumulating Ito cells, which are characterized by collagen synthesis, were very often found (Figure 9). These are elongated stellate cells, localized most often within the Disse space, but they were also often detected in the connective tissue stroma of the liver lobules, especially near the portal tracts. With a strong accumulation of lipids, their large drops displaced the nucleus to the periphery of the cell, thereby changing its shape. In addition to lipid droplets, many cisterns of the granular endoplasmic reticulum were characteristically detected in the cytoplasm of these cells.

**Figure 10:**
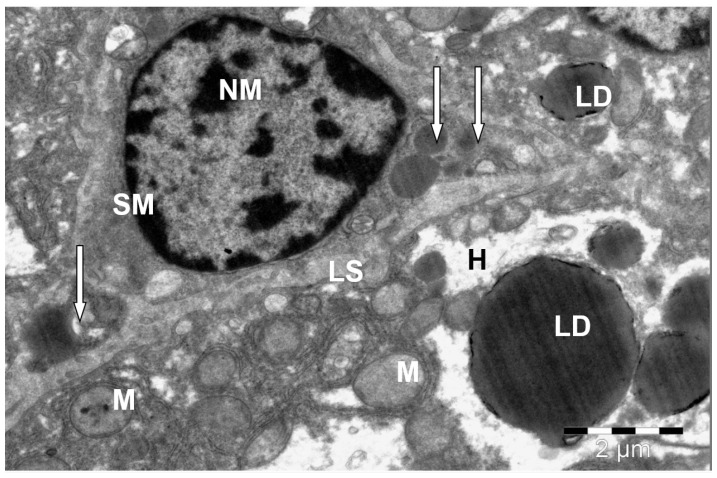
Stellate macrophage (Kupffer cell) in the liver of a camel. H—hepatocyte; SM—stellate macrophage; NM- macrophage nucleus; LS—sinusoid lumen; LD—lipid droplets; M—mitochondria; lysosomes and phagolysosomes (↑). Electron microphotography.

Large dendritic cells with high phagocytic activity, stellate macrophages (Kupffer cells) (Figure 10), were found in the lumens of sinusoidal capillaries. The number of these cells was 15–20 cells per 10,000 µm^2^. They had one large nucleus of an elongated oval or irregular shape, sometimes with strongly jagged edges. A large amount of heterochromatin was detected in the nuclei, which either lay on the internal karyolemma or was located diffusely in large clumps throughout the nuclear matrix.

These cells were anchored with their basal ends in the bifurcations between hepatocytes, while their bodies and processes lay freely in the lumen of the sinusoidal capillary.

**Figure 11:**
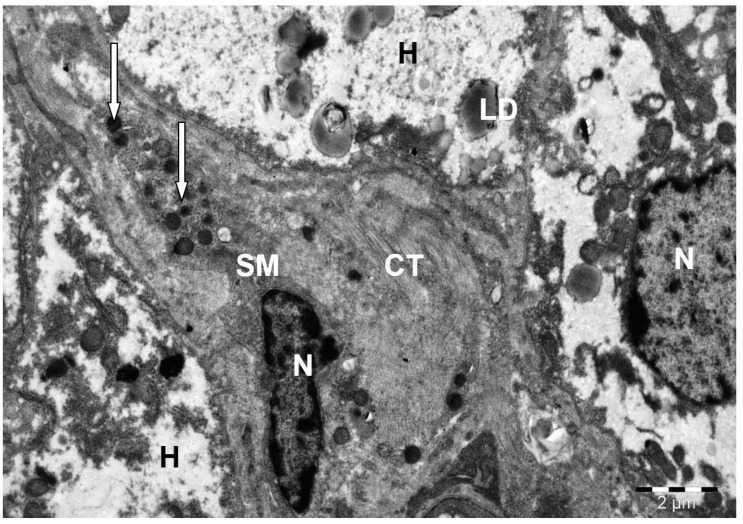
Connective tissue (CT) strands and a stellate macrophage (Cooper cell) (SM) in the lumen of a dilated sinusoidal capillary of camel liver. H—hepatocyte; N—nucleus; L—lipid inclusions; M—mitochondria; lysosomes and phagolysosomes (↑). Electron microphotography.

This type of cell was characterized by electron-dense cytoplasm, which was due to the high content of ribosomes, polyribosomes, small granules and vesicles, lysosomes, and heterogeneous phagolysosomes. Their cytoplasm could also contain many small, rounded mitochondria, a well-developed Golgi complex, and short cisterns of the granular endoplasmic reticulum. Sometimes, parts of degraded erythrocytes and hemosiderin inclusions were found in the cytoplasm. Also, in many areas of the liver parenchyma, along with the presence of stellate macrophages, many sinusoidal capillaries were filled with strands of connective tissue (Figure 11).

## 4. Discussion

Unlike other mammals, the camel’s liver exhibits a number of characteristics that can be linked to its lifestyle and metabolic requirements [2,8,9].

Firstly, the special morphological characteristics of the hepatocytes indicate a high capacity for metabolism and detoxification [24,25]. Their large size may be related to the need to store metabolites (glycogen) and water for drought tolerance. The narrow sinusoids observed in the liver may facilitate efficient interactions between hepatocytes and Kupffer cells, which are critical for immune defense and metabolism in settings where parasitic and microbial infections are highly likely. Higher thickness of the Disse space ensures mechanical strength and protection under volumetric loads on the organ.

The number of Ito cells is not increased, but they are in a more activated state. These are the cells responsible for collagen production, so the thickening of connective tissue indicates their active role in maintaining structural integrity and not pathological fibrosis [26,27].

Increased density of the Kupffer cells (15–20 cells per 10,000 µm^2^ versus 10–15 in other species) provides immune protection in conditions of potentially increased bacterial load from the gastrointestinal tract when consuming roughage and quickly drinking large volumes of water [12,28,29,30].

Secondly, the observed increase in the amount of fat deposits in hepatocytes may be a sign of adaptation to periods of food shortage. It may also indicate a role for the liver in regulating energy metabolism and accumulating nutrient reserves necessary for survival in arid conditions [31,32,33]. A small number of hepatocytes with lipid droplets prevents lipotoxicity and fatty liver degeneration under metabolic stress. In the camel, a predominance of small and medium lipid droplets (0.5–2 µm in diameter) is expected, while in animals prone to steatosis, large droplets (over 5 µm) are also found, which merge and disrupt cell function [12,14,28,34].

Key differences in the microstructural organization of camel hepatocytes are associated with adaptation to life in an arid environment and consist of the ultrastructural features of their organelles. Mitochondria are larger and more numerous with a dense matrix for increased energy efficiency in fat metabolism (the main source of energy and metabolic water) [35]. The endoplasmic reticulum (especially the smooth reticulum) in camels is more developed for enhanced detoxification and protein synthesis, and peroxisomes have increased activity for alternative oxidation pathways, which overall provides the unique ability of the camel liver to function effectively under conditions of prolonged dehydration and metabolize large volumes of fat and decay products [35,36].

Our studies highlight the great diversity of bactrian liver structure and raise important questions about the influence of various environmental factors on its morphology. These ultrastructural features are directly related to the environmental conditions of camels, namely, the need to effectively withstand prolonged dehydration and metabolic stress in the desert. The reduced content of lipid droplets in hepatocytes (12–15%) is an adaptation to minimize the risk of steatosis and maintain high metabolic activity of the liver under stress, while the increased thickness of connective tissue (1.2–1.5 μm) provides mechanical stability and protection of hepatocytes from damage during fluctuations in organ volume caused by rapid consumption of large volumes of water. From a physiological point of view, this determines the high resistance of camels to hepatosis and fibrosis, and from a veterinary point of view, it means the need to revise the standard diagnostic criteria (for example, blood biochemical parameters) for this species of animals, since their liver has a unique morpho-functional organization. These results can form the basis for further research in the field of comparative anatomy and physiology, as well as for assessing the health and productivity of camels in pasture and farm animal husbandry conditions.

It is also worth noting that the specificity of the liver microstructure may be useful for veterinary practice, in particular for the development of preventive measures against liver diseases in camels. Understanding the liver’s unique adaptations may help develop less invasive diagnostic and therapeutic methods, which in turn may improve the care of these animals [37,38,39].

This study sheds light on important aspects of the microstructural organization of the bactrian liver and highlights the need for further study of this topic to better understand the adaptive mechanisms that allow this species to thrive in extreme conditions.

## 5. Conclusions

The ultrastructural organization of the camel liver demonstrates a complex of adaptations aimed at maintaining metabolic stability and mechanical strength under extreme conditions. Low lipid content, enhanced collagen scaffold, and, presumably, an active macrophage system work synergistically to prevent dehydration and stress-related damage. This detail makes the camel liver an extremely resistant organ compared to the liver of domestic animals.

The analysis of the liver microstructure also opens up new horizons for studying diseases of this animal, which can contribute to the development of more effective diagnostic and therapeutic methods. The study of the microstructural organization of the Bactrian camel liver emphasizes the importance of the adaptation of this species to its habitat and opens up new perspectives for studying the functional biology and ecology of camels in general.

## Data Availability

Data is contained within the article. The original contributions presented in this study are included in the article. Further inquiries can be directed to the corresponding author.

## Abbreviations

AgER	agranular endoplasmic reticulum channels
CF	collagen fibers
CT	connective tissue
E	bile duct epithelial cells
EP	endothelial cell processes
Er	erythrocyte
F	fibroblast cells
GC	Golgi complex
GER	granular endoplasmic reticulum channels
H	hepatocyte
HC	hepatocyte cytoplasm
L	lipid inclusions
LBD	lumen of the bile duct
LD	lipid droplet
LS	sinusoid lumen
LV	lumen of the central vein
M	mitochondria
N	nucleus
NI	Ito cell nucleus
NM	macrophage nucleus
NN	nucleolus in the nucleus
R	ribosomes and polyribosomes
SL	sinusoid lumen
SM	stellate macrophage
V	hepatocyte villi
